# Micro-CT evaluation of internal fit of two different implant-abutment connections with different customized abutment materials

**DOI:** 10.1186/s12903-025-06837-y

**Published:** 2025-09-18

**Authors:** Ahmed Ziada, Marwa Beleidy

**Affiliations:** 1 Department of Fixed Prosthodontics, Faculty of Dentistry, Benisuef University, Benisuef, Egypt; 2 Department of Fixed Prosthodontics, Faculty of Dentistry, October 6th University, Giza, Egypt

**Keywords:** Hybrid connection, Star-shaped tube-in-tube connection, CAD/CAM abutments, Soft-milled Co-Cr-Mo, Zirconia, Titanium, And implant-abutment interface

## Abstract

**Background:**

The internal fit of the implant-abutment connection plays a crucial role in implant success. This study aims to assess the impact of various connection designs and materials of customized implant abutments on the gap distance at the implant-abutment interface.

**Methods:**

Two internal connection implant systems, star-shaped tube-in-tube and hybrid Morse taper with internal hex anti-rotation feature (*n* = 24 each, total *n* = 48) were evaluated. Each group was subdivided into four material groups: zirconia (Zr), titanium (Ti), cobalt-chromium (Co-Cr), and soft-milled cobalt-chromium-molybdenum (Co-Cr-Mo). The internal fit was assessed via micro-computed tomography (µCT).

**Results:**

All specimens demonstrated clinically acceptable microgap values (≤ 150 μm). The star-shaped tube-in-tube connection exhibited significantly narrower gaps compared to the hybrid connection. Zr showed the greatest gaps among materials, followed by Ti, Co-Cr, and soft-milled Co-Cr-Mo, which had the smallest gaps.

**Conclusions:**

The star-shaped tube-in-tube connection demonstrated superior internal fit to the hybrid connection. Zirconia abutments demonstrated inferior internal adaptability. Dimensional changes during sintering of soft milled materials may have influenced these outcomes and should be considered when interpreting the data. All tested abutments fell within clinically acceptable misfit ranges.

## Background

In dental treatment, two-piece implant systems, which include the implant and an abutment, are commonly used. A significant drawback of this system is the unavoidable formation of microgaps resulting from the misalignment at the implant-abutment interface (IAI). Dental implant manufacturers have focused on optimizing implant–abutment connections to enhance sealing and reduce peri-implant tissue inflammation [1, 2].

Implant–abutment connections are classified as external or internal. External connections, typically involving an external hex interface, position the abutment on top of the implant platform and are generally associated with greater susceptibility to microgaps and sequent bone loss [3]. Diverse geometries of internal connections have been presented, including internal hexagon, internal octagon, conical, and tube-in-tube configurations, which produce a cold-welded connection characterized by high friction [4, 5]. Compressive loads lead to increased abutment settling, thereby reducing the microgap and allowing the two structures to function as a one unit, minimizing microleakage and improving rotation and bending torque resistance [6].

Prefabricated abutments provide satisfactory fit but frequently result in suboptimal emergence profiles, necessitating extensive prosthetic contouring [4]. Custom abutments have been proposed to enhance soft tissue adaptation and esthetics. However, their clinical use is limited due to potential casting inaccuracies, suboptimal fit, reduced rotational stability, and the rising cost of noble metal alloys [7, 8]. The computer-aided design and computer-aided manufacturing (CAD/CAM) abutments enable the replication of the emerging contours of individual teeth, facilitate the precise construction of the final prosthesis, and improve retention and support, resulting in reliable outcomes and biocompatibility [9].

Considerations such as abutment construction material, design, and surface topography affect IAI microgap and soft tissue preservation [10, 11]. Titanium (Ti) is commonly used for stock and customized abutments, showing similar vertical gaps at the IAI [12–15]. However, biocompatibility, strength, and aesthetics make zirconia (Zr) a popular aesthetic ceramic material for implant prosthetic abutments and crowns [16, 17]. With gold prices increasing, cobalt-chromium (Co-Cr) makes customized abutments at a lower cost than precious alloys. Also, CAD/CAM Co-Cr reduces human errors, time, and costs while providing a clean and precise surface finish [18]. An alternative method for producing customized Co-Cr abutments involves milling a wax-like textured pre-sintered disc, which enables the dry milling of the material. However, they do not assert that implant connections can be formed this way.

Various methods, such as sectioning, scanning electron microscopy (SEM), optical microscopy, and X-ray microcomputed tomography (µCT), are used to measure the microgap at IAI [19–22]. µCT is especially effective due to its high resolution (0.5 μm), 3D imaging, and non-invasive nature, allowing precise measurement of length, area, and volume [23, 24]. It also supports the analysis of ferromagnetic materials.

This research aimed to examine the internal fit at the IAI across two distinct internal implant-abutment connection geometries; a star-shaped tube-in-tube and a hybrid Morse taper with internal hex anti-rotation feature representing two of used but structurally different design philosophies in implant dentistry and four materials of customized implant abutments. The first null hypothesis posited that a nonsignificant microgap difference would exist between the two implant-abutment systems. The second null hypothesis was that the customized abutment materials would demonstrate no significant differences.

## Methods

The protocol for this study was approved by the Faculty of Dentistry Beni-Suef University Research Ethics Committee (FDBSU-REC) (No. REC-FDBSU/0212025-03/ZA).

### Sample size calculation

Utilizing alpha (α) and beta (β) values of 0.05, which correspond to 95% statistical power and an effect size (f) of 0.333 obtained from prior research, the minimum requisite sample size (n) was determined to be 6 specimens for each group [25]. The sample size was computed using a sample size calculation software (G*Power 3.1.9.7 for Windows, HHUD, Germany).

### Specimens’ preparation

Based on the type of connection, 48 implants were utilized in this study, with 24 implants in each group. Two groups of connections were presented: Group (S) with a 6-point star-shaped tube-in-tube connection (Torx^®^; Classic Sky, Bredent GmbH, Germany) and Group (H) with a hybrid 5° Morse taper and an internal hex anti-rotation feature connection (Conexa; EvLine, B&B Dental S.r.l, Italy). Each group was divided into four subgroups based on the manufacturing material (*n* = 6): TitaniumTi Grade V (Ti) (imes-icore GmbH, Eiterfeld, Germany), Zirconia (Zr) (Ceramill Zolid HT, Ammann Girrbach, Austria), cobalt-chromium (Co-Cr) (Remanium Star MD II, Dentaurum, Germany), and soft-milled cobalt-chromium-molybdenum (Co-Cr-Mo) (Ceramill Sintron, Ammann Girrbach, Austria).

In order to simulate osseointegration and ensure consistent stabilization during micro-CT scanning, a dental surveyor (B2 Parallelometer; Bio-Art Soluções Inteligentes, São Carlos, Brasil) was used to position the implants longitudinally in a vertical alignment of the implant long axis inside polypropylene tubes (PP-R pipe PN16 SDR7.4; EGIC, Cairo, Egypt) that were 25.0 mm in diameter and 20.0 mm length, filled with dual polymerizing acrylic resin (LuxaCore^®^ Dual, DMG, Hamburg, Germany). Following ISO 14801:2016, a three-millimeter space was created between the implant shoulder and the top surface of the tube to simulate a clinical scenario where the implant platform is positioned above the bone level, as can occur with bone remodeling or resorption. This approach minimized specimen movement and allowed reproducible µCT cross-sectional imaging across all specimens.

**Fig. 1:**
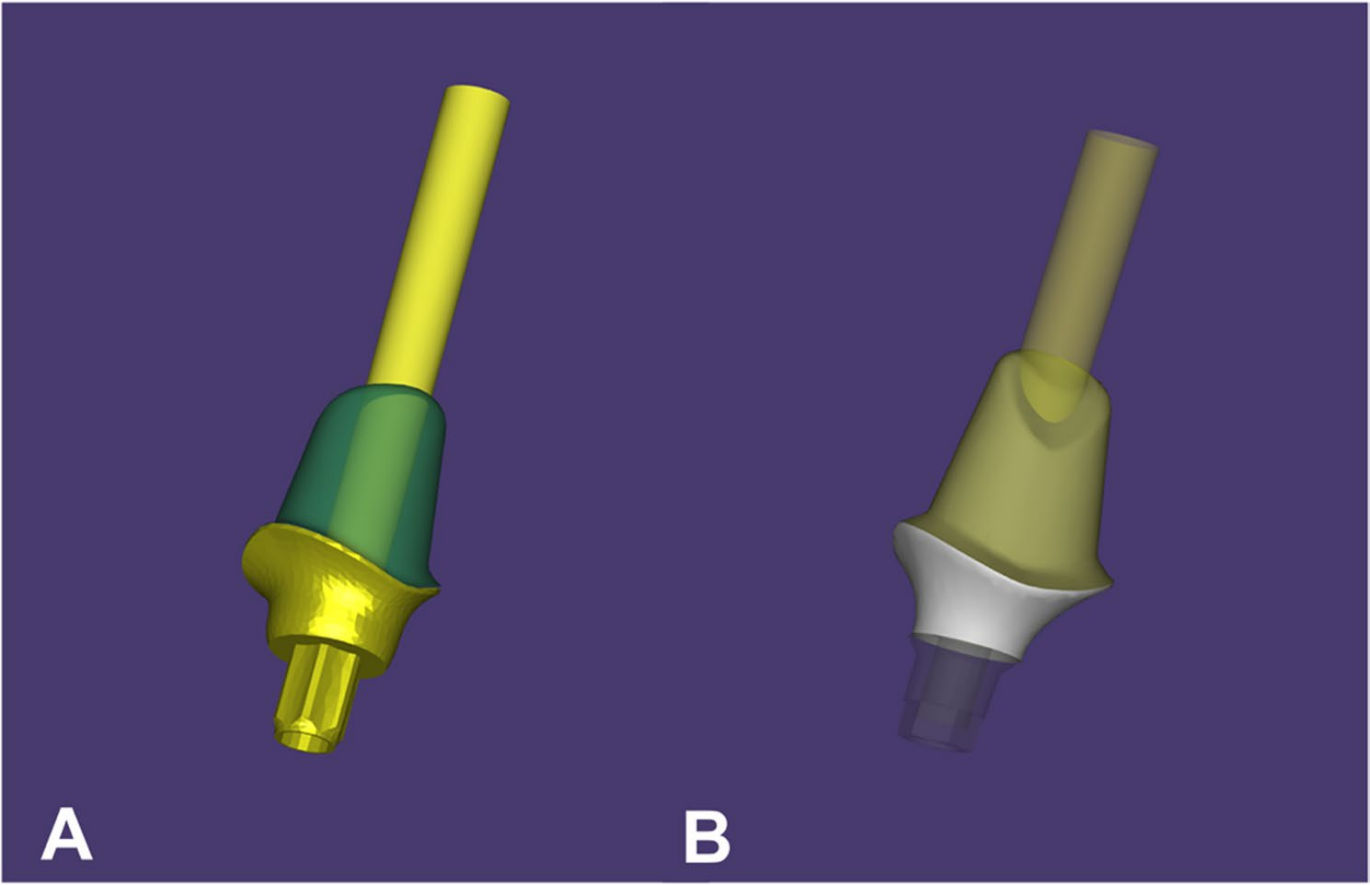
Abutments’ designing with a star shaped tube-in-tube connection (**A**) and with a hybrid (conical-hex design) connection (**B**) using CAD software

The abutment design for group (S) was initiated using CAD software (exocad DentalCAD v3.0; exocad GmbH, Darmstadt, Germany) with a star-shaped tube-in-tube connection (Fig. 1A). A duplicate design with a hybrid Morse taper with internal hex anti-rotation feature connection was created for group (H) using scan bodies attached to one implant from each group and scanned to determine the implant position (Fig. 1B). Both sorts of connections were obtained from the software library. The software employs a built-in compensation algorithm for both Zr and Co-Cr-Mo abutments to account for anticipated sintering shrinkage.

**Fig. 2:**
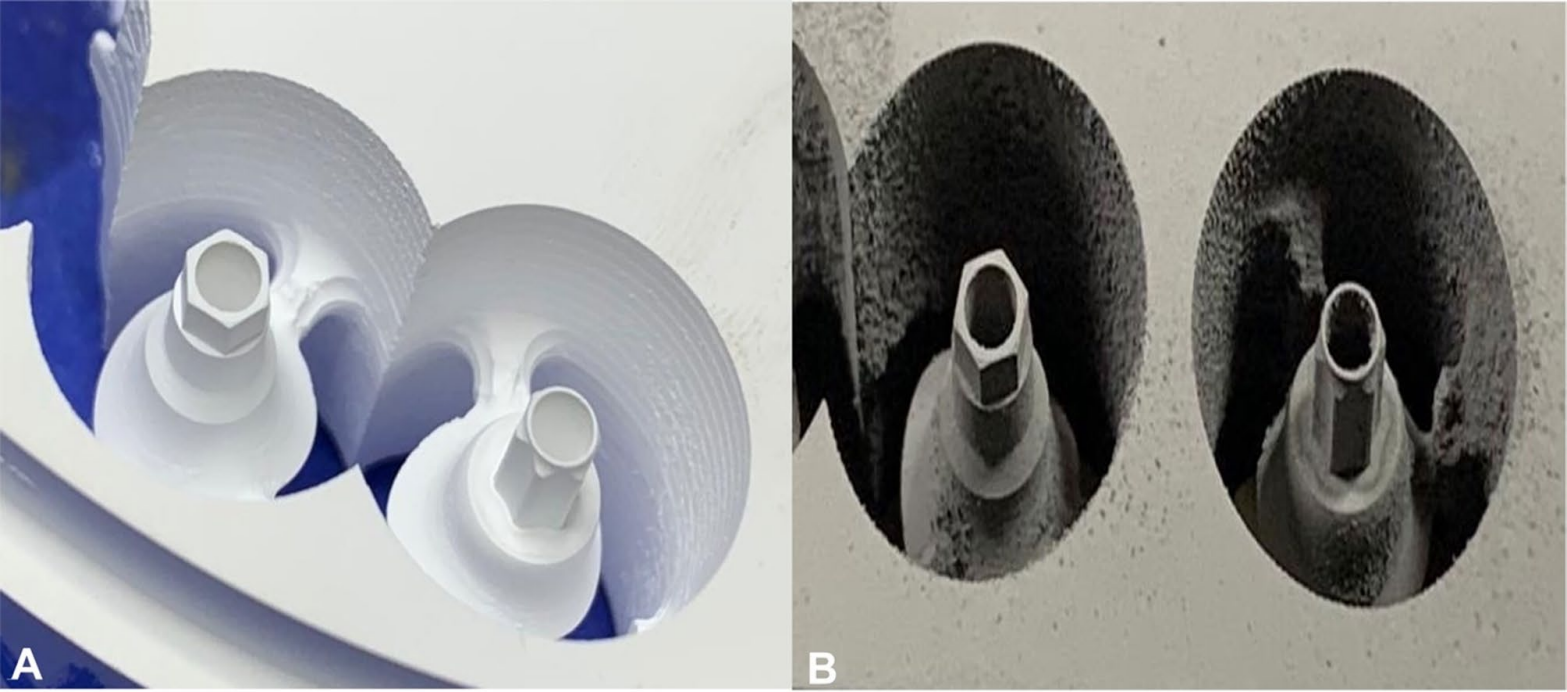
5-axis CNC milling of designed abutments into distinct materials, Zirconia (**A**) and soft-milled Co-Cr-M (**B**)

Each design was produced using a 5-axis CNC milling machine (ED5X; Emar, Arsharqia, Egypt) (Fig. 2) from the four distinct materials (Ti, Zr, Co-Cr and Co-Cr-Mo). Following milling, the Zr, and Co-Cr-Mo abutments were subjected to sintering in furnaces (Ceramill Therm 3 and Ceramill Argotherm 2, Ammann Girrbach, Austria) following the manufacturers’ specifications.

**Fig. 3:**
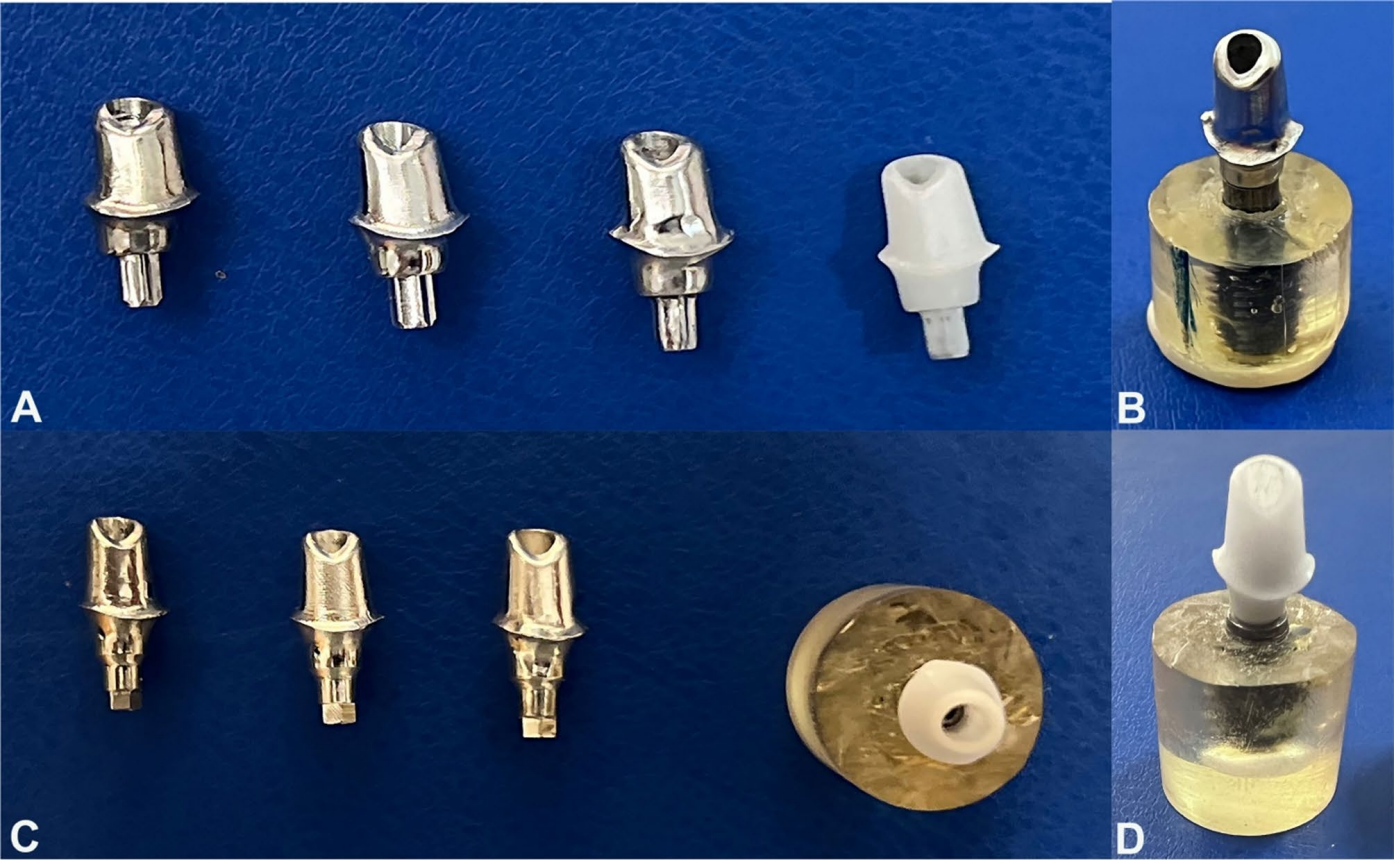
Different implant abutment designs and materials after manufacturing. Group S abutments in four different materials (**A**), screwed S abutment on its corresponding implant (**B**), Group H abutments in four different materials (**C**) and screwed H abutment on its corresponding implant (**D**)

The abutments (Fig. 3) were then secured to their implants using the manufacturer’s recommended torque of 25 N⋅cm, applied using a digital torque gauge (TQ 680; TORQUÍMETRO DIGITAL PORTÁTIL, São Paulo, Brazil), which has an accuracy of 0.1 N⋅cm.

#### µCT measurement

Following calibration with a standard 5-ruby artifact, all specimens were scanned using a micro-computed tomography system (Nikon MCT 225; Nikon Metrology, Tring, UK). Scanning parameters were set to a voxel size of 9.2 μm, acceleration voltage of 170 kV, filament power of 7.6 W, and an exposure time of 4000 ms. A 250 μm aluminum filter was employed to reduce beam hardening artifacts. Specimens were mounted vertically in a custom-designed holder to ensure consistent orientation and minimize motion-related artifacts.

**Fig. 4:**
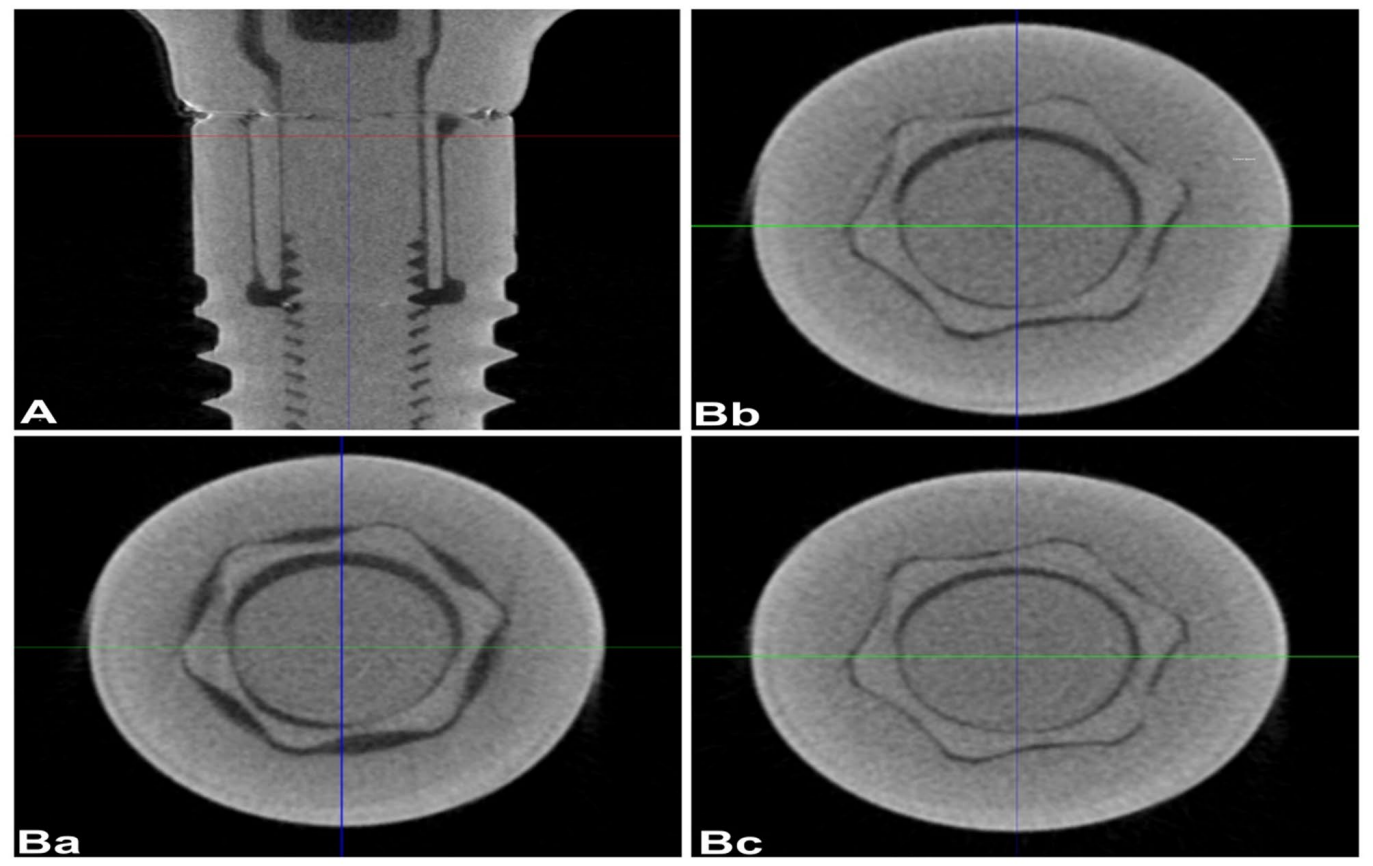
3D reconstructed µCT vertical (**A**) and horizontal (**B**) section images of star shaped connection indicating the top **(Ba**), middle (**Bb**) and bottom (**Bc**) sections

**Fig. 5:**
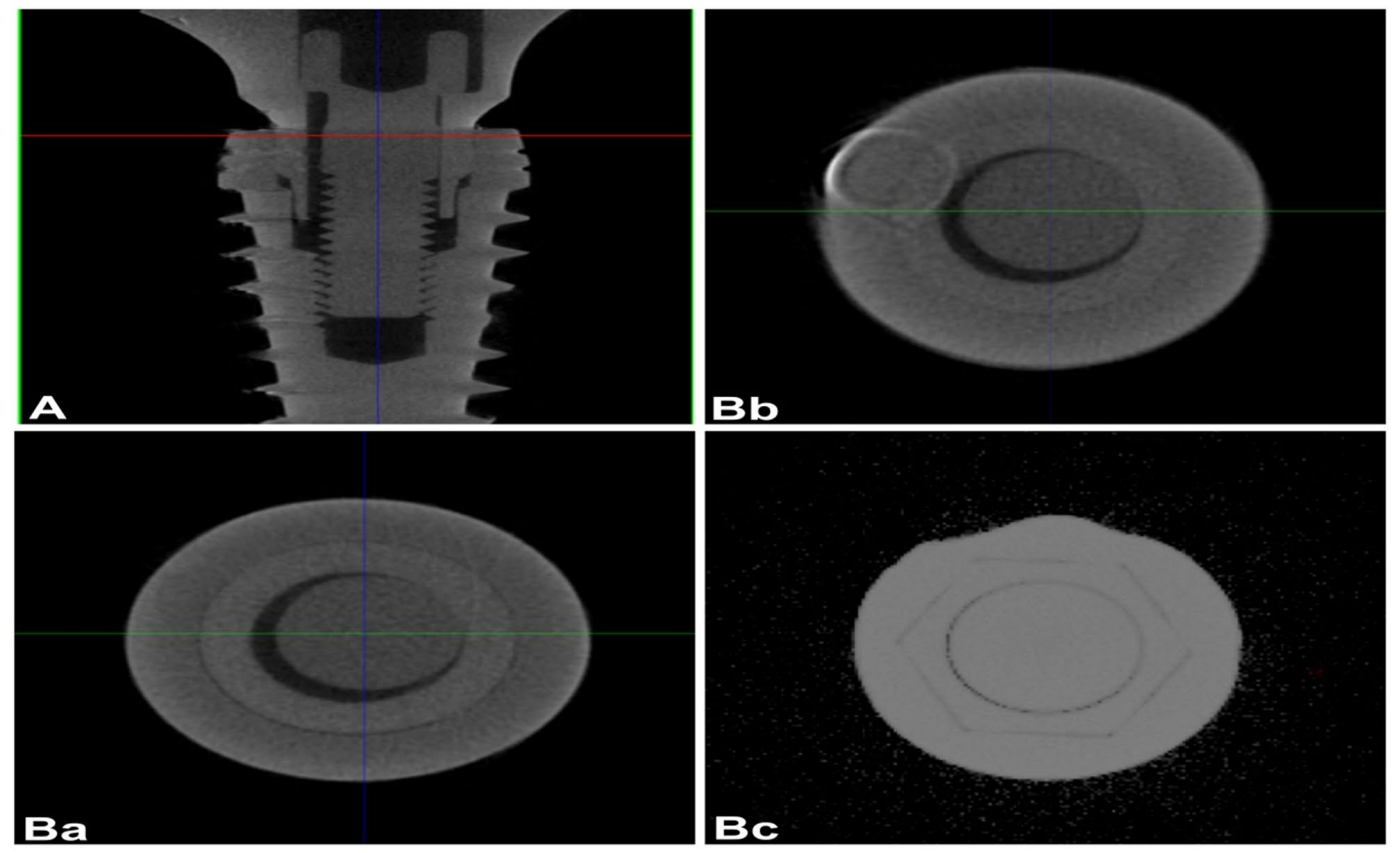
3D reconstructed µCT vertical (**A**) and horizontal (**B**) section images hybrid connection indicating the top (**Ba**), middle (**Bb**) and bottom (**Bc**) sections

Reconstructed 3D images (Figs. 4 and 5) were processed using a 3D simulation and analysis software (VG Studio MAX 3.4; Volume Graphics GmbH, Heidelberg Germany). Digital correction algorithms were applied for precise segmentation and analysis of the implant–abutment interface.The 3D data were shown in horizontal and sagittal slices to evaluate the internal fit with the help of the software’s linear measurement tool. A horizontal plane with marked 7 points was set as a reference. To ensure consistent analysis across specimens, seven geometrically defined points were established along the implant–abutment interface based on the total vertical length (L) measured along the implant’s longitudinal axis. These included the interface boundaries at 0 (point 1), L/3 (point 2), 2 L/3 (point 3), and L (point 4), which defined the Top, Middle, and Bottom regions. Within each region, a centralized cross-sectional point was identified to represent the coronal, middle, and apical thirds: specifically, L/6 (point 5) for the Top, L/2 (point 6) for the Middle, and 5 L/6 (point 7) for the Bottom. Although only three regions were analyzed, the use of these sevenstandardized coordinate points ensured precise and repeatable slice selection from the µCT dataset, enabling accurate comparison across all implant–abutment specimens.Three sections perpendicular to the reference plane, top, middle, and bottom, were defined through marked points (Figs. 4 and 5), and the internal gap length was measured in each section. A step-by-step specimen measurement of a cross-sectional layer image was obtained from the constructed 3D scan. Ten equidistant points were used for the measurements, which were calculated with the Measurement-3D distance Length tool value in microns (µm). The µCT result analysis was carried out with a fixed grey value threshold of ISO 50% for all the scans and used for all the specimens by a single operator. All measurements were taken at a magnification of 100×, and each measurement was repeated three times, and the mean value was used.

### Statistical analysis

The distribution of numerical data was shown by calculating the mean and standard deviation (SD). While checking the distribution, Shapiro-Wilk’s test was used to ensure normality, and Levene’s test was used to ensure variance homogeneity. The data followed a normal distribution, and the variances of the various variables were similar. First, a three-way mixed model ANOVA was used to assess them. Then, the error term of the multifactorial model was used to compare simple effects. The False Discovery Rate (FDR) was used to modify the p-values for multiple comparisons. All tests were conducted with a significance threshold of *p* < 0.05. Software for statistical analysis (R, Ver. 4.4.2 for Windows; CRAN, Veinna, Australia) was used for the analysis.

## Results

The three-way mixed model ANOVA findings indicated a significant interaction among the three examined factors for internal fit (*p* < 0.001), as shown in Table 1. The findings from simple effects comparisons indicated that irrespective of measurement section location and abutment material, internal gaps assessed with the star-shaped tube-in-tube connection were significantly less than those of the hybrid connection, as observed in Table 2.

**Table 1:** Results of three-way mixed ANOVA of different interactions of variables (implant-abutment connection, abutment material and section location)

Source	Sum of squares (III)	df	Mean square	f-value	*p*-value
Connection	2524.45	1	2524.45	505.79	< 0.001*
Material	644.01	3	214.67	43.01	< 0.001*
Section	3209.21	2	1604.60	1099.57	< 0.001*
Connection * Material	284.88	3	94.96	19.03	< 0.001*
Connection * Section	478.10	2	239.05	163.81	< 0.001*
Material * Section	327.21	6	54.54	37.37	< 0.001*
Connection * Material * Section	323.55	6	53.92	36.95	< 0.001*

*df* degree of freedom, * significant (*p*<0.05)

**Table 2:** The mean, standard deviation (SD) values and results of repeated measures ANOVA test for comparison between internal fit (µm) in different sections with different interactions of variables (connection and abutment material)

Section	Connection	Internal fit (µm) (Mean ± SD)			
		Zr	Ti	Co-Cr	Co-Cr-Mo
Top	**Star shaped (S)**	48.50 ± 2.00^A^	36.00 ± 2.65^B^	31.00 ± 1.00^C^	34.00 ± 2.00^BC^
	**Hybrid (H)**	52.83 ± 2.02^A^	53.33 ± 1.04^A^	51.67 ± 1.04^A^	54.11 ± 0.10^A^
*p*-value		0.002*	< 0.001*	< 0.001*	< 0.001*
Effect size	**Eta squared (95% CI)**	0.237 (0.057:0.409)	0.833 (0.732:0.877)	0.876 (0.799:0.909)	0.870 (0.790:0.904)
Middle	**Star shaped (S)**	48.50 ± 2.00^A^	37.00 ± 0.00^B^	31.00 ± 1.00^C^	24.50 ± 0.00^D^
	**Hybrid (H)**	54.17 ± 3.51^A^	50.11 ± 0.96^B^	47.33 ± 2.75^B^	50.83 ± 0.76^AB^
*p*-value		< 0.001*	< 0.001*	< 0.001*	< 0.001*
Effect size	**Eta squared (95% CI)**	0.347 (0.137:0.506)	0.740 (0.594:0.809)	0.816 (0.705:0.864)	0.920 (0.869:0.941)
Bottom	**Star shaped (S)**	27.50 ± 2.00^A^	27.00 ± 0.00^A^	28.50 ± 1.00^A^	28.00 ± 2.00^A^
	**Hybrid (H)**	34.67 ± 1.15^A^	30.89 ± 0.77^B^	32.33 ± 1.44^AB^	31.33 ± 1.26^AB^
*p*-value		< 0.001*	0.006*	0.007*	0.017*
Effect size	**Eta squared (95% CI)**	0.460 (0.243:0.597)	0.200 (0.036:0.374)	0.196 (0.034:0.369)	0.156 (0.016:0.328)

Values with different superscripts within the same horizontal row are significantly different, * significant (*p*<0.05), ns not significant

No significant difference was noted in the adaptation of various materials for top measurements using H connection and bottom measurements employing the S connection. In top measurement using the S connection, internal gaps assessed with Zr were markedly larger than those tested with other materials. Moreover, the gaps seen in Ti abutments were significantly greater than those in Co-Cr. All material comparisons were statistically significant in the middle measurements using the S connection, with Zr exhibiting the largest gaps, followed by Ti, Co-Cr, and Co-Cr-Mo. In middle measures using the H connection, gaps assessed with Zr abutments were considerably bigger than those evaluated with Ti and Co-Cr. In bottom measurements of the H connection, the gaps in Zr abutments were markedly greater than those in Ti abutments. However, no significance was noted in the S connection, as shown in Table 3.

**Table 3:** The mean, standard deviation (SD) values and results of repeated measures ANOVA test for comparison between internal fit (µm) with different section locations

Connection	Material	Internal fit (µm) (Mean ± SD)			*p*-value	Effect size
		Top	Middle	Bottom		Eta squared (95% CI)
Star shaped (S)	**Zr**	48.50 ± 2.00^Aa^	48.50 ± 2.00^Aa^	27.50 ± 2.00^Ba^	< 0.001*	0.950 (0.913:0.962)
	**Ti**	36.00 ± 2.65^Ab^	37.00 ± 0.00^Ab^	27.00 ± 0.00^Ba^	< 0.001*	0.938 (0.894:0.954)
	**Co-Cr**	31.00 ± 1.00^Ac^	31.00 ± 1.00^Ac^	28.50 ± 1.00^Ba^	0.022*	0.796 (0.660:0.847)
	**Co-Cr-Mo**	34.00 ± 2.00^Abc^	24.50 ± 0.00^Cd^	28.00 ± 2.00^Ba^	< 0.001*	0.950 (0.913:0.962)
p-value		< 0.001*	< 0.001*	0.702		
Effect size	**Eta squared (95% CI)**	0.855 (0.753:0.887)	0.912 (0.848:0.931)	0.040 (0.000:0.111)		
Hybrid (H)	**Zr**	52.83 ± 2.02^Aa^	54.17 ± 3.51^Aa^	34.67 ± 1.15^Ba^	< 0.001*	0.211 (0.018:0.369)
	**Ti**	53.33 ± 1.04^Aa^	50.11 ± 0.96^Bb^	30.89 ± 0.77^Cb^	< 0.001*	0.930 (0.879:0.947)
	**Co-Cr**	51.67 ± 1.04^Aa^	47.33 ± 2.75^Bb^	32.33 ± 1.44^Cab^	< 0.001*	0.748 (0.586:0.811)
	**Co-Cr-Mo**	54.11 ± 0.10^Aa^	50.83 ± 0.76^Bab^	31.33 ± 1.26^Cab^	< 0.001*	0.951 (0.916:0.963)
p-value		0.326	< 0.001*	0.034*		
Effect size	**Eta squared (95% CI)**	0.095 (0.000:0.206)	0.440 (0.185:0.557)	0.220 (0.010:0.355)		

Values with different Capital superscripts within the same horizontal row are significantly different, while Values with different Small superscripts within the same vertical culum are significantly different, * significant (*p*<0.05)

The results indicated a notable disparity in measurements taken at various section locations, irrespective of connection type and abutment material. In specimens featuring star-shaped tube-in-tube connections and soft-milled Co-Cr abutments, all comparisons yielded statistically significant results, with the greatest gaps seen at the top, followed by the bottom, and the smallest gaps in the middle. For other materials exhibiting the same connection, the measurements at the top and middle were much superior to those at the bottom, as shown in Tables 2 and 3.

The top and middle section measures were markedly greater than the bottom values for specimens, including the hybrid connection and zirconia abutments. All comparisons exhibited statistical significance for other materials with the same connections, with the greatest gaps recorded at the top, followed by the middle, and the smallest gaps observed at the bottom, as shown in Table 3.

## Discussion

In the present study, two distinct internal implant-abutment connection designs representing different engineering philosophies concerning mechanical engagement, load distribution, and sealing potential. The star-shaped tube-in-tube connection offers a more unified geometry, combining a Torx-like anti-rotational feature with a cylindrical mating surface. This design promotes more centralized force transmission and improved seating consistency, potentially enhancing mechanical stability and microbial sealing as claimed by the manufacturer. In contrast, the hybrid connection, which typically includes a conical interface coupled with an anti-rotational hex component, is widely used due to its ease of alignment and resistance to rotational forces [26, 27]. The selection of these two geometrically distinct systems allowed for a meaningful comparison of how connection morphology influences the accuracy of fit, particularly when combined with different abutment materials.

Recognizing the inherent platform heterogeneity between the two evaluated implant systems is important. Differences in design tolerances, proprietary manufacturing processes, and material handling protocols are intrinsic to commercially available implant systems and may have influenced the observed outcomes. Although such variability introduces confounding factors, it also reflects real-world clinical scenarios where clinicians must choose among systems with differing engineering philosophies.

The advancement of CAD/CAM technology has significantly accelerated digital dentistry and expanded the selection of materials used in implant prosthodontics. CAD/CAM abutments allow for precise customization, combining the mechanical reliability of stock abutments with the flexibility to modify key prosthetic parameters such as emergence profile, angulation, and finish line location [14, 28, 29]. Despite these advancements, limited data exist on the accuracy of fit for customized abutments made from different materials. Although gaps at the IAI are considered inevitable [20, 30], achieving an optimal fit with minimal microgaps remains essential for clinical success and should guide implant component design and fabrication [31].

Methodological inconsistencies across studies, ranging from sample size and the number of measurements to the measuring technique, contribute to variations in reported implant-abutment fit outcomes [32–40]. µCT proved effective for non-destructive, three-dimensional evaluation of implant–abutment fit, allowing repeated measurements without sample damage [41, 42]. Its superior resolution compared to traditional methods enabled detailed analysis across multiple sites. Future improvements in the software may enhance quantitative assessment of contact areas, improving understanding of interface integrity and clinical relevance.

The described adequate gap between the prosthetic abutment/framework and the implant has been varying over time, ranging between 30 μm and 150 μm [20, 43–45] Currently, there is a shortage of conformity to the question of “What is the acceptable gap between implant and abutment?” [46]. Various studies considered a tolerable gap distance should not exceed 49 μm [47, 48]. Considering those studies, the gap distance of all studied specimens in the present study was within an acceptable range. However, other reports described the acceptable gap to be less than 10 μm [49–51].

Consistent results across both groups led to rejecting the first null hypothesis. The star-shaped tube-in-tube connection demonstrated significantly smaller mean internal gap distances than the hybrid connection (*p* < 0.001), indicating superior internal fit. This can be attributed to its uniform interlocking geometry, which promotes extensive surface contact, self-alignment, and predictable seating [52]. In contrast, the hybrid connection demonstrates complex design and intersecting geometries increasing the risk of misalignment, tolerance stacking, and incomplete seating, contributing to larger internal gaps [53]. While limited literature specifically addresses the internal fit performance of star-shaped tube-in-tube connections, the present study addresses this gap by evaluating the fit characteristics of this design. The findings align with previous research on trilobe (tube-in-tube) connections, which reported significantly smaller microgaps and reduced bacterial leakage compared to internal hex designs, supporting the underlying design philosophy of improved sealing capability [54].

A comprehensive examination of the mean values in the Top, Middle, and Bottom sections revealed that although subgroups exhibited the highest mean values in the Top section and the lowest mean values at Bottom section among all specimens. The apical region of the star-shaped tube-in-tube connection showed better fit than the coronal portion. This could be due to the self-centering geometry guide the abutment more precisely as it seats deeper, ensuring tighter contact apically that might be further enhanced by the application of tightening torque [52]. Additionally, the apical area typically experiences greater compressive forces during torque application, which helps the components settle and adapt more closely. In the hybrid connection, a partial adaptation was observed at the top portion of the conical interface; a more accurate fit was noted in the anti-rotational hexagonal region of the hybrid connection. The internal hex may interrupt the taper’s sealing continuity, slightly compromising fit consistency compared to the more uniform tube-in-tube geometry in Group S. Abutments might fail to fully seat within their designated implant positions due to premature contact at the apical region of the internal connection. This is particularly problematic in the absence of a defined butt margin or reference stop, even with a clinically acceptable gap at the IAI. Such incomplete engagement could occur despite applying the manufacturer’s recommended torque, suggesting underlying fabrication inconsistencies such as tolerance stacking or inconsistencies in material removal. One potential cause is variability in the seating behavior of components, which might be influenced by technical limitations inherent to the milling process used in fabricating CAD/CAM abutments. In particular, milling machines might struggle to replicate the precise geometry of the 5° Morse taper, which features a complex combination of conical angles and rounded internal transitions. This could lead to angular mismatches or oversized contact zones that prevent intimate contact along the taper surface, compromising fit. Tool-geometry mismatch between the milling bur and the implant’s internal configuration, especially at curvature transitions, might further exacerbate seating discrepancies. These factors might collectively contribute to misfit at IAI, which may impact sealing efficacy [55, 56]. This aligns with the findings of Queiroz et al. [57], who concluded that variations in abutment materials and fabrication techniques could lead to differing degrees of misfit at the implant-abutment interface. These observations highlight that taper angle alone does not determine sealing ability; the manufacturing accuracy, material stiffness, and connection depth also play critical roles.

However, in the absence of direct assessments such as torque–angle analysis, surface deviation mapping, or optical inspection of mating surfaces, these interpretations remain speculative and warrant further investigation.

The second null hypothesis regarding the abutment material’s influence on microgap at IAI was rejected. Zirconia demonstrated the poorest performance among all tested materials, regardless of the connection type, exhibiting the highest mean internal gap values. This was followed by titanium, while soft-milled Co-Cr-Mo and Co-Cr presented the smallest gap values, indicating that abutment material significantly influences the IAI fit.

Importantly, titanium and Co-Cr were milled from fully sintered blocks. In contrast, zirconia and Co-Cr-Mo were milled in a partially sintered (soft) state and subjected to high-temperature sintering. Although soft milling allows for easier and more precise shaping, the subsequent sintering causes volumetric shrinkage and dimensional changes that can negatively impact the internal fit. Zirconia undergoes significant sintering shrinkage reported to be up to 20% of its original volume which likely accounts for the larger internal gaps observed [58]. The current findings were consistent with Barbosa Jr. et al., who reported that titanium abutments had a superior fit (11.1 μm) compared to zirconia abutments (25.3 μm) in external hex connections [14]. Similarly, Queiroz et al. noted increased wear and misfit in customized zirconia abutments, raising concerns about long-term IAI stability [57] . Baldassarri et al. demonstrated significantly better adaptation in titanium abutments than zirconia across various implant systems with conical connections [59]. Additionally, Molinero-Mourelle et al. reported no significant difference in angular misfit between milled Co-Cr and zirconia conical abutments compared to cast and laser-sintered Co-Cr. However, overall performance was better for milled components [34].

Soft-milled Co-Cr-Mo abutments exhibited less sintering shrinkage (approximately 10%, as indicated by the manufacturer) than zirconia, which may contribute to the improved fit observed in this study [60, 61]. However, Ramalho et al. found that soft-milled Co-Cr-Mo abutments had higher misfit volumes than machined and cast abutments [36]. Such discrepancies may arise from differences in abutment connection designs, measurement methodologies, or whether marginal or internal adaptation was assessed.

Although CAD/CAM systems apply shrinkage compensation algorithms, no dimensional verification was performed in the current study to confirm abutment accuracy. Variations in geometry, material batches, or furnace conditions might have caused unmeasured deviations, potentially affecting internal fit and introducing errors in the results.

### Limitations

This study focused on implant abutment internal fit, but mechanical stability, micromovements at the implant-abutment interface, and long-term biomechanical performance also affect implant-supported restoration clinical success [62, 63]. The current study focused on dimensional analysis under standard conditions, hence these factors were not examined. Nevertheless, the internal gap remains an important parameter influencing mechanical integrity and microbial sealing.

However, this study addressed additional problems. The absence of a control group representing a gold standard abutment-implant interface with known optimal fit parameters (e.g., a one-piece milled titanium abutment with the original manufacturer’s implant system) is a one limitation. Such a control would have provided a reliable benchmark to contextualize the microgap values observed across experimental groups. Only two internal implant-abutment connections were examined; more designs and bigger sample sizes might offer more comprehensive results. Microgap measurements were done using µCT, but implementing other techniques like SEM and measuring before and after load application would provide more insights. This in vitro study does not replicate key intraoral conditions such as occlusal loading, saliva, microbial activity, or tissue response, underscoring the need for clinical studies. No direct measurements (e.g., post-milling dimensional analysis, surface deviation scans, or comparison to design files) were conducted to verify seating completeness of implant components or manufacturing deviations as post-sintering shrinkage of zirconia and soft-milled Co-Cr-Mo abutments, therefore results should be evaluated cautiously.

Future high-resolution metrology research must isolate sintering effects on fit and prove clinical relevance.Additionally, studies should integrate assessments of functional loading, fatigue behavior, and interface micromotion to comprehensively evaluate implant–aututment system performance. Utilizing standardized reference platforms or custom-manufactured components in future research may further isolate the specific effects of connection geometry from manufacturing variability.

## Conclusions

The star-shaped tube-in-tube connection demonstrated superior internal fit to the hybrid connection, with more consistent results across all tested materials. CAD/CAM zirconia abutments demonstrated inferior internal adaptability across the two connections used, while Titanium, Chromium Cobalt, or soft milled Chromium Cobalt possess better internal fit. Dimensional changes during sintering, especially for zirconia and soft-milled Co-Cr-Mo, may have influenced these outcomes and should be considered when interpreting the data. All tested abutments fell within clinically acceptable misfit ranges.

## Data Availability

The datasets used and/or analysed during the current study are available from the corresponding author on reasonable request.
